# Hepatitis C virus attributable liver cancer in the country of Georgia, 2015–2019: a case–control study

**DOI:** 10.1186/s12879-024-09916-7

**Published:** 2024-09-27

**Authors:** Sophia Surguladze, Paige A. Armstrong, Geoff A. Beckett, Shaun Shadaker, Amiran Gamkrelidze, Maia Tsereteli, Vladimer Getia, Benedict Oppong Asamoah

**Affiliations:** 1 Task Force for Global Health, Tbilisi, Georgia; 2 Centers for Disease Control and Prevention, Atlanta, USA; 3 National Center for Disease Control and Public Health, Tbilisi, Georgia; 4 Division of Social Medicine and Global Health, Department of Clinical Sciences Malmö, Lund University, Malmö, Sweden

**Keywords:** Viral hepatitis, Hepatitis C, Liver cancer, Hepatocellular carcinoma, Georgia

## Abstract

**Background:**

Hepatitis C virus (HCV) infection can lead to a type of primary liver cancer called hepatocellular carcinoma (HCC). Georgia, a high HCV prevalence country, started an HCV elimination program in 2015. In addition to tracking incidence and mortality, surveillance for the HCV-attributable fraction of HCC is an important indicator of the program’s impact. This study assesses HCV infection-attributable HCC in the Georgian population.

**Methods:**

This case–control study utilized HCV programmatic and Georgian Cancer Registry data from 2015–2019. Bivariate logistic regression and age- and sex-stratified analyses assessed HCV and liver cancer association. HCV-attributable liver cancer proportions for the HCV-exposed and total population were calculated. A sub-analysis was performed for HCC cases specifically.

**Results:**

The total study population was 3874 with 496 liver cancer cases and 3378 controls. The odds for HCV-infected individuals developing liver cancer was 20.1 (95% confidence interval [CI] 15.97–25.37), and the odds of developing HCC was 16.84 (95% CI 12.01–23.83) compared to the HCV-negative group. Odds ratios varied across strata, with HCV-infected older individuals and women having higher odds of developing both liver cancer and HCC. A large proportion of liver cancer and HCC can be attributed to HCV in HCV-infected individuals; however, in the general population, the burden of liver cancer and HCC cannot be explained by HCV alone.

**Conclusion:**

HCV was significantly associated with a higher risk of developing liver cancer and HCC in the Georgian population. In addition, given Georgia’s high HCV burden, increased HCC monitoring in HCV-infected patients is needed.

## Introduction

Hepatitis C virus (HCV) is a blood-borne virus that targets the liver, resulting in acute and chronic infection, and can cause liver cirrhosis and hepatocellular carcinoma (HCC). The World Health Organization (WHO) estimates that in 2019, an estimated 58 million individuals worldwide were chronically infected with HCV [1]. The HCV-attributable proportion of HCC varies globally [2], however, an estimated 20% of all global liver cancer cases are attributable to HCV infection [3].

Georgia, a country of 3.7 million people, was found to have a high HCV prevalence according to a 2015 nationwide cross-sectional seroprevalence study [4]. The study found that 7.7% of the Georgian adult population had evidence of exposure to HCV (anti-HCV antibody positive) and 5.4% had chronic HCV infection (HCV RNA positive); the most significant risk factors were reported history of injection drug use and blood transfusion [4]. Due to the high disease burden, Georgia initiated a nationwide HCV elimination program in 2015. Together with the United States Centers for Disease Control and Prevention and Gilead Sciences (which donated direct-acting antiviral medications), the Georgian government set the goal of screening the entire adult population and providing free treatment to all those with chronic HCV infection [5–8]. The program aimed to identify 90% of adults infected, treat 95% of those diagnosed, and cure 95% of those treated [9, 10]. Individuals can be screened for anti-HCV at testing sites throughout the country in a variety of settings (including public spaces like town halls), undergo mandatory screening when admitted to hospitals, and be tested at antenatal clinics, and blood donation centers, among other locations [7]. Testing is also available for high-risk population groups such as persons who inject drugs (PWID), persons experiencing incarceration, and patients on hemodialysis [5–7, 11, 12]. Integrated screening services for HCV, HIV, and tuberculosis are also offered at some locations [5–7, 11, 12]. Anti-HCV-positive patients are referred for viremia testing at designated facilities, and if positive, are referred for clinical evaluation and treatment [5–7]. A patient is considered to be cured when they achieve sustained virologic response (SVR) 12 weeks after completing treatment [5–7]. As of November 2019, over 56% (1,594,269 individuals) of the Georgian adult population had been screened for anti-HCV, of whom over 120,000 were anti-HCV positive [13]. Over 81,000 individuals were diagnosed with chronic HCV infection, around 64,000 initiated treatment, and among those tested for SVR, 98.9% achieved cure.^1^

A technical advisory group (TAG) was established as part of the program, comprised of international experts in the field [9, 10, 14]. Since 2015, the TAG has recommended strengthening surveillance of HCV-attributable diseases and specifically, HCC [10].

Limited data are available to conclusively estimate HCC-attributable HCV cases in Georgia, however, the country has robust HCV elimination program data and a national population-based cancer registry that can be linked, allowing for a comprehensive assessment of the impact of HCV infection on liver cancer in the Georgian population. This study aimed to assess HCV infection-attributable HCC in a country with high HCV prevalence.

## Materials and methods

### Study design

This case–control study was based in the country of Georgia. The study period included all liver cancer cases during 2015–2019 in the Georgian Cancer Registry (GCR). The year 2015 was chosen as the baseline as that is when the HCV elimination program began and the HCV screening and treatment data collection and monitoring came into full effect. Due to the SARS-CoV-2 pandemic during the data extraction period and the subsequent lag in data retrieval and analysis from the relevant databases, the data cut-off was determined to be the year 2019 as the newest data available at the time was up to 2020.

### Data sources

The databases used for this study were the national HCV screening registry and treatment databases and the GCR; all databases are owned by the Georgian government. Every Georgian citizen is assigned an 11-digit unique ID at birth and these IDs are used to link individual-level case data between the databases.

The national HCV screening registry and HCV treatment databases, created in 2017 and 2015 respectively, contain information on demographics, screening, viremia, and other diagnostic testing, HCV genotype, treatment initiation and completion, and treatment results (including retrospective hepatitis C screening data) [12]. The screening database also contains the screening results of those individuals tested for HCV before the elimination program began which was inputted into the database in 2015 and the screening data of those screened between 2015 and 2017.

The GCR was established in 2015 by the Georgian National Centre for Disease Control and Public Health (NCDC) and includes new, confirmed, and suspected physician-diagnosed cancer cases, following standards and methods recommended by the World Health Organization International Agency for Cancer Research as well as demographic data, detailed clinical, radiological, and received services data, and international classification of disease (ICD-10) classification codes [15].

### Participant selection

**Fig. 1:**
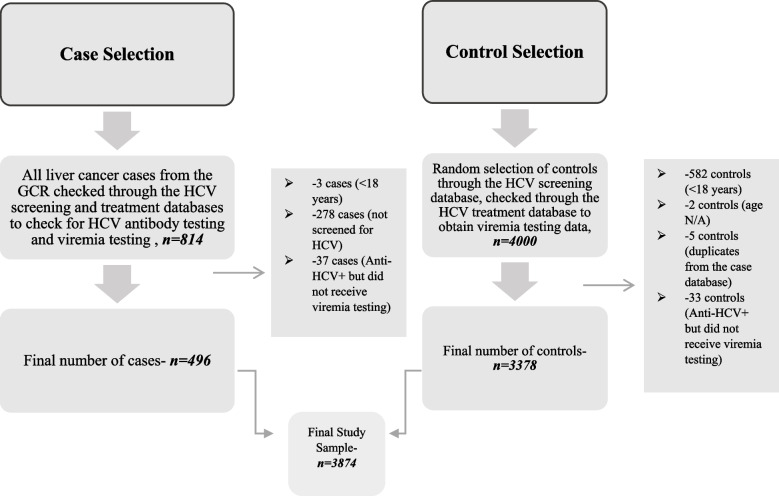
Case and control selection from the Georgian population for the years 2015–2019. *Note:* All liver cancer cases as well as random controls were selected from the years 2015–2019 from the Georgian Cancer Registry and the Hepatitis C Screening Database respectively. *Abbreviations:* HCV = hepatitis C virus, Anti-HCV = antibodies against hepatitis C virus, GCR = Georgian Cancer Registry

Figure 1 is a flow diagram showing the case and control selection process. A case was defined as an adult individual (≥ 18 years of age) who appeared in the GCR with any primary cancer of the liver or intrahepatic bile duct cancer ICD-10 code (C22.0–C22.9) who also had been screened for anti-HCV, and, in the case of screening positive, had viremia test results available. A control was defined as an adult individual who did not appear in the GCR with any ICD-10 C22 code and who was screened for HCV and if screened positive, received viremia testing.

The cases of liver cancer were identified by applying selection criteria to all recorded liver cancer cases in the GCR during 2015–2019. The full range of classifications used encompasses all malignant neoplasms of the liver and intrahepatic bile ducts; this can include hepatocellular carcinoma, intrahepatic cholangiocarcinoma, and other specified and unspecified liver cancers [16]. The decision to include all liver cancer ICD-10 codes and not only the HCC-specific ICD-10 code was due to limitations in diagnosis and the likelihood that HCC cases were classified as liver cancer, and to ensure that all possible HCC cases were included. Liver cancer is diagnosed at late stages in Georgia when a biopsy is not feasible or useful for patients in hospice care – such patients are typically coded as unspecified primary or secondary liver cancer [17]. Furthermore, a previous Georgian HCC analysis showed inconsistencies with ICD-10 codes between the physician-coded diagnoses and the GCR ICD-10 codes [17] making the ICD-10 code classifications not entirely reliable. The decision was made to include all liver cancer cases in the main analysis and perform a sub-analysis of the HCC-specific ICD-10 code C22.0 to assess the results.

As the study took all liver cancer cases as cases and then applied selection criteria, we decided to randomly pick 4000 controls to have at least a 1:5 case-to-control ratio. The randomly selected controls were then compared with cases to avoid duplication. This database was used for random selection as it could act as a good proxy for the general adult population and it covered more than 56% of the Georgian adult population in 2019; hepatitis C screening is widely available and adopted by the general population in Georgia [18].

The total study sample size came to 3874, with 496 cases and 3378 controls, for a case-to-control ratio of 1:7.

### Variables and measurement

The outcome variable in this study was liver cancer. As previously mentioned, the ICD-10 code submitted to the GCR is sometimes modified and changed according to the medical histories of the patients; for this study, the final GCR-assigned ICD-10 codes were chosen to assess outcome status as they are more reliable and are fully available for every case, unlike the original ICD-10 codes from the medical records. The liver cancer variable was coded in 2 different ways for the analysis; the first version of the variable contains all cases with ICD-10 codes for hepatobiliary cancer diagnoses (ICD-10 C22.0–C22.9) as cases and the second version contains only those with the ICD-10 C22.0 code for the HCC sub-analysis with the rest of participants with other ICD-10 codes (C22.1-C22.9) considered as controls.

The exposure variable in this study was chronic HCV infection. Participants were exposed if they tested positive for anti-HCV and viremia (HCV RNA or core antigen). Those with a negative screening test or with a positive screening test but a negative HCV viremia test were considered as HCV negative. Those with anti-HCV positive results but no viremia testing were excluded from the general analysis. However, to solidify the analysis and check for sensitivity, two other scenarios were taken into account during the analysis; the first scenario assumed that those without HCV viremia testing but a positive screening test had chronic HCV and the second scenario assumed that they did not. Thus, two other subsets were created where HCV exposure was coded by either including the ambiguous cases as HCV positive or negative. This was done for the overall liver cancer group only.

The demographic variables analyzed in this study for confounding and effect modification were age and sex. The age of the participants was categorized as follows: 18–35 years, 36–49 years, 50–61 years, and > 61 years; the 36–49 age group was selected as the reference category in the analysis, as the youngest group had a very small fraction of liver cancer cases and using it as a reference category would decrease the statistical power of the models.

### Statistical methods

Statistical analyses for this study were performed using the R statistical software with statistical significance determined using an alpha of 0.05. Both bivariate and multivariable logistic regression models were used to calculate odds ratios (OR) and their 95% confidence intervals (95% CI). The fit of the models was accounted for by the Akaike information criterion (AIC) [19].

The first bivariate logistic regression was performed with chronic HCV as the exposure and liver cancer (with all C22 ICD-10 codes included) as the outcome. Next, stratified bivariate logistic regression analyses were run for age and sex to check the variability of the ORs across the strata and consequently check for confounding by observing whether the crude OR of the initial model was in the ranges of the 95% CIs generated by the bivariate stratified analyses and also as a first step, to check for effect modification by observing the variability across strata specific ORs. Afterward, as a second step for checking for effect modification, a multiplicative logistic regression model was run to check for significant interaction between age and HCV, and sex and HCV; statistical significance was determined at the P < 0.05 threshold. HCV-attributable liver cancer proportion among the HCV-exposed population (APe) and HCV-attributable liver cancer proportion among the total population (APt) were calculated for age and sex strata individually using each stratum-specific OR and for the overall study population. The same analysis was performed for the HCC sub-group.

APe and APt were calculated using the following formulas, where OR refers to the odds ratios and Pe refers to the proportion of the population with the exposure [20]:

$$\text{APe}=[(\text{OR}-1)/\text{OR}] *100$$

$$\text{APt}= [\text{Pe }* ((\text{OR}-1)/\text{OR}))] *100$$

## Results

Table 1 shows that in the total study population (cases and controls) the prevalence of chronic HCV infection was 11.5%. Overall, 50.6% were male and a plurality (30.0%) were aged 18–35 years. In the liver cancer analysis, the prevalence of chronic HCV was 53.2% among cases and 5.4% among controls, and in the HCC analysis, the prevalence was 63.3% among cases and 9.3% among controls. The majority of liver cancer cases were among those aged > 61 years (49.2%) and the majority of HCC cases were aged 50–61 years (46.2%). The lowest proportion of liver cancer and HCC cases were among the youngest age group (18–35 years), at 0.8% and 1.3% respectively. Three-quarters of both liver cancer and HCC cases were in males.

**Table 1:** Frequency of hepatitis C, liver cancer, hepatocellular carcinoma in the georgian sample population (*n* = 3874), and prevalence of liver cancer and hepatocellular carcinoma (*n* = 3874) by hepatitis C status, age, and sex, 2015–2019

*A*	*Overall Frequency*	*%*
**Hepatitis C virus Infection**		
Yes	445	11.5%
No	3429	88.5%
Total	3874	100%
**Sex**		
Male	1959	50.6%
Female	1915	49.4%
Total	3874	100%
**Age (years)**		
18–35	1161	30.0%
36–49	808	20.9%
50–61	842	21.7%
> 61	1063	27.4%
Total	3874	100%
***B***	***Liver Cancer (All ICD-10 codes for hepatobiliary cancer diagnoses included), n*** ** = ** ***496, N (%)***	***No Liver Cancer N (%)***
**Hepatitis C**		
Positive	264 (53.2%)	181 (5.4%)
Negative	232 (46.8%)	3197 (94.6%)
**Age (years)**		
18–35	4 (0.8%)	1157 (34.3%)
36–49	43 (8.7%)	765 (22.6%)
50–61	205 (41.3%)	637 (18.9%)
> 61	244 (49.2%)	819 (24.2%)
**Sex**		
Females	124 (25.0%)	1791 (53.0%)
Males	372 (75.0%)	1587 (47.0%)
***C***	***Hepatocellular Carcinoma (Only hepatocellular carcinoma-specific ICD-10 code—C22.0 -included), n*** ** = ** ***158, N (%)***	***No Hepatocellular Carcinoma N (%)***
**Hepatitis C**		
Positive	100 (63.3%)	345 (9.3%)
Negative	58 (36.7%)	3371 (90.7%)
**Age (years)**		
18–35	2 (1.3%)	1159 (31.2%)
36–49	16 (10.1%)	792 (21.3%)
50–61	73 (46.2%)	769 (20.7%)
> 61	67 (42.4%)	996 (26.8%)
**Sex**		
Females	38 (24.1%)	1877 (50.5%)
Males	120 (75.9%)	1839 (49.5%)

For most cases of HCC and HCV infection, diagnosis of HCV preceded HCC diagnosis; for 39 cases, HCV diagnosis followed HCC diagnosis. However, in all 39 cases, the HCV diagnosis was made within three months of the HCC diagnosis.

Table 2 shows that in bivariate analysis, those with chronic HCV infection were 20.01 times more likely to develop liver cancer than those without infection (95% CI 15.95–25.37), and those with chronic HCV were 16.84 times more likely to develop HCC than those without (95% CI 12.01–23.83).

**Table 2:** Results of the bivariate logistic regression models- looking at the association between hepatitis C and liver cancer and hepatitis C and hepatocellular carcinoma and results of the sensitivity analyses^a^, Georgia, 2015–2019

**Bivariate Logistic regression for Liver Cancer Analysis and Hepatocellular Cancer Sub-analysis**				
	***General liver cancer analysis bivariate regression model (All ICD-10 codes for hepatobiliary cancer diagnoses included): AIC***^b^ ***2303***		***Hepatocellular sub-analysis bivariate regression model (Only hepatocellular carcinoma-specific ICD-10 code—C22.0 -included): AIC 1066***	
	Frequency (n)	OR^c^ (95% CI^d^)	Frequency (n)	OR (95% CI)
Hepatitis C negative	3429	ref^e^	3429	ref
Hepatitis C positive	445	20.01 (15.97 –25.37)	445	16.84 (12.01–23.83)
**Sensitivity Analyses (Based on General Liver Cancer Analysis)**				
	***Bivariate logistic Regression- All hepatitis C screened individuals with positive test results without a viremia test considered hepatitis C positive (AIC 2400)***		***Bivariate logistic Regression- All hepatitis C screened individuals with positive test results without a viremia test considered hepatitis C positive (AIC 2502)***	
	Frequency (n)	OR (95% CI)	Frequency (n)	OR (95% CI)
Hepatitis C negative	3429	ref	3499	ref
Hepatitis C positive	515	19.38 (15.57–24.19)	445	17.51 (13.98–21.99)

^a^Bivariate logistic regression models looking at the association between hepatitis C and general liver cancer in the years 2015–2019 in which either all individuals positive for hepatitis C antibodies without a confirmatory test are considered to be hepatitis C positive or all are considered to be hepatitis C negative

^b^Akaike information criterion

^c^Odds Ratio

^d^95% Confidence Interval

^e^Reference category

Table 2 also depicts the results of the sensitivity analyses which checked for the association between HCV and liver cancer in which anti-HCV positive individuals without viremia testing (*n* = 70) were considered as either HCV positive or negative. When including them as HCV-positive, the odds ratio was 19.38 (95% CI 15.57–24.19), and in the model that considered these cases as negative, the odds ratio was 17.51 (95% CI 13.98–21.99).

Table 3 depicts the results of the bivariate analyses examining the association between HCV and liver cancer and HCC stratified by age group and sex. In age-stratified analyses, when compared to their HCV-negative counterparts, the odds of having liver cancer was lowest among those aged 18–35 years (OR 10.69; 95% CI 0.52–85.84) and highest among those aged 50–61 years (OR 27.84; 95% CI 18.54–42.53). When stratified by sex, the odds ratio for females was found to be 25.76 (95% CI 15.09–44.45) and for males 14.84 (95% CI 11.39–29.43). The crude OR for liver cancer of 20.01 is within the range of age-stratified and sex-stratified odds ratios, thus, neither age nor sex appears to be confounders in the relationship between HCV and overall liver cancer. A high variation in estimates was also observed in the HCC sub-analysis across both the age and sex strata, with the youngest age group and women with HCV having the highest odds of developing HCC. Age and sex also were not found to be confounders in the relationship between HCV and HCC. All observed ORs in the age and sex-stratified analyses were statistically significant for both analyses.

**Table 3:** Results of bivariate logistic regression models—looking at the association between hepatitis C and liver cancer and hepatitis C and hepatocellular carcinoma stratified by age and sex, Georgia, 2015–2019

***Liver Cancer Analysis (All ICD-10 codes for hepatobiliary cancer diagnoses included)***						
	Age strata (Years)				Sex	
	18 – 35 OR^a^ (95% CI^b^)	36 –49 OR (95% CI)	50 –61 OR (95% CI)	> 61 OR (95% CI)	Male OR (95% CI)	Female OR (95% CI)
Hepatitis C negative	ref^c^	ref	ref	ref	ref	ref
Hepatitis C positive	10.69 (0.52–85.84)	19.94(10.25–40.52)	27.84(18.54–42.53)	20.44 (12.64–34.36)	14.84 (11.39–29.43)	25.76 (15.09–44.45)
***HCC Sub-Analysis (Only hepatocellular carcinoma-specific ICD-10 code-C22.0- included)***						
	Age strata (Years)				Sex	
	18 – 35 OR (95% CI)	36 –49 OR (95% CI)	50 –61 OR (95% CI)	> 61 OR (95% CI)	Male OR (95% CI)	Female OR (95% CI)
Hepatitis C negative	ref	ref	ref	ref	ref	ref
Hepatitis C positive	32.11 (1.25–823.3)	12.84 (4.6–38.52)	12.33 (7.22–21.95)	13.43(7.88–23.02)	12.26 (8.43–29.3)	24.35(11.78–49.18)

^a^Odds Ratio

^b^95% Confidence Interval

^c^Reference category

Table 4 shows the results of the multivariable models checking for effect modification for the age and sex variables in both the liver cancer analysis and the HCC sub-analysis. No statistically significant interaction effect was detected between HCV and age, nor between HCV and sex in the liver cancer analyses (*P* = 0.86 and 0.07 respectively). Similarly, there was no statistically significant interaction in the HCC sub-analyses.

**Table 4:** Results of multivariate logistic regression models checking for age and sex as effect modifiers for the relationship between hepatitis C infection and liver cancer and hepatitis C and hepatocellular carcinoma, Georgia, 2015–2019

	**Liver cancer analysis bivariate regression model (All ICD-10 codes for hepatobiliary cancer diagnoses included):**	**Hepatocellular sub-analysis bivariate regression model (Only hepatocellular carcinoma-specific ICD-10 code-C22.0- included):**
**Checking for Age as an Effect Modifier**		
	OR^a^ (P-value)	OR (P-value)
**Hepatitis C**		
Negative	ref^b^	ref
Positive	21.68 (1.18e-11)	12.95 (6.7e-05)
**Age (Years)**		
18–35	0.11 (6.03e-05)	0.15 (0.015)
36–49	ref	ref
50–61	5.73 (9.77e-11)	3.44 (0.001)
> 61	10.17 (< 2e-16)	4.13 (0.001)
**Interaction term**		
Hepatitis C*Age	1.03 (0.859)	1.00 (0.988)
**Checking for Sex as an Effect Modifier**		
	OR (P-value)	OR (P-value)
**Hepatitis C**		
Negative	ref	ref
Positive	25.76 (< 2e-16)	24.30 (< 2e-16)
**Sex**		
Female	ref	ref
Male	2.01 (5.83e-07)	1.80 (0.0294)
**Interaction term**		
Hepatitis C* Sex	0.58 (0.0721)	0.52 (0.1189)

^a^Odds Ratio

^b^Reference category

Table 5 depicts HCV-attributable liver cancer and HCC proportions in both the HCV-exposed and the total population, for the overall study population and stratified by age and sex. The Ape for the overall study population was 95.0% and the Apt was 11.3%. The APe and APt calculations also differed from each other across the age and sex strata. Among those aged 18–35 years with a history of chronic HCV, 90.6% of liver cancer cases can be attributed to HCV; in the general population of this age group, 2.8% of liver cancer cases can be attributed to HCV infection. On the other hand, in the 50–61 age group the APe and APt were found to be 96.4% and 23.5% respectively. The same was found for sex; in men with a history of chronic HCV, 92.3% of liver cancer cases can be attributed to HCV, compared to 18.1% of males in the total population. Among females, the APe was 96.1% and the APt was 2.8%.

**Table 5:** Age and sex stratified hepatitis C-attributable liver cancer and hepatitis C-attributable hepatocellular carcinoma proportion calculations in the hepatitis C exposed population and in the total population, Georgia, 2015–2019

*Liver Cancer Analysis (All ICD-10 codes for hepatobiliary cancer diagnoses included)*							
	Age (years) Strata				Sex Strata		Overall APe (APt)
	18–35 APe^a^ (APt^b^)	36–49 APe (APt)	50–61 APe (APt)	> 61 APe (APt)	Males APe (APt)	Females APe (APt)	95.0% (11.4%)
Hepatitis C positive	90.6% (2.8%)	95.13% (11.9%)	96.4% (23.5%)	95.1% (10.1%)	92.3% (18.1%)	96.1% (2.8%)	
***HCC Sub-Analysis (Only hepatocellular carcinoma-specific ICD-10 code-C22.0- included)***							
	Age (years) Strata				Sex Strata		Overall APe (APt)
	18–35 APe (APt)	36–49 APe (APt)	50–61 APe (APt)	> 61 APe (APt)	Males APe (APt)	Females APe (APt)	94.1% (11.3%)
Hepatitis C positive	96.8% (3.0%)	92.2% (11.5%)	91.8% (22.1%)	92.5% (9.5%)	91.9% (17.8%)	92.5% (3.5%)	

^a^Attributable proportion in exposed population

^b^Attributable proportion in the total population

For the HCC sub-analyses, the APe and APt calculations for age and sex strata showed similar results to the liver cancer analyses.

## Discussion

This study found that chronic HCV infection was significantly associated with higher odds of developing liver cancer as well as HCC specifically in the Georgian population. This effect differs by age and sex, with older populations and women with chronic HCV infection experiencing higher odds of subsequent disease diagnosis. Generally, in the cases, diagnosis of HCV preceded HCC diagnosis; suggesting that HCV was a pre-existing condition and diagnosed as part of the evaluation for HCC. The study also found that while HCV-attributable liver cancer and HCC proportions are high in the overall study population and among the strata in the exposed population, the HCV-attributable proportions are generally lower in the total population.

In the sensitivity analyses the ORs were similar to those derived from cases with documented viremia status with overlapping 95% CIs, indicating that the original model was not highly sensitive to the exclusion of HCV ambiguous cases. Despite the fact that no statistically significant interactions were found when checking for effect modification, the varied ranges in ORs across the age and sex strata indicate potential effect modification in both analyses. However, this study did not analyze effect modification using the additive scale.

Other studies conducted on this topic report similar findings. The high magnitude of effect of HCV on the development of liver cancer and the observed phenomenon of older age increasing the risk of HCC is a common finding. A case–control study in West Africa found that HCV-infected individuals had a 35.9 times higher risk of developing HCC than those without infection [21]. Another US study on elderly patients found that those aged ≥ 66 years with HCV had a higher risk of liver cancer [22]. A second US study calculated the HCV-attributable HCC proportions in the total population and found that metabolic disorders, hepatitis B virus (HBV), HCV infection, smoking, alcohol consumption, and genetic disorders were all significant predictors of HCC in the population [23], highlighting that many other predictors of HCC exist. Furthermore, when combining all known HCC predictors, the US study found that they explained around 60% of HCC cases in the country, leaving the possibility of other unknown factors ^23^.

In our study, females had a higher risk of being diagnosed with liver cancer and HCC than males among those with HCV infection. This is in contrast with other studies that have shown that men are more predisposed to HCC than women [24–26]. These results could suggest certain sex-related disparities relating to the development of HCC after HCV infection or could derive from the fact that generally women have higher life expectancies than men and survive long enough to develop cancer. Furthermore, while men are thought to be more highly predisposed to developing HCC, older women are more likely to be screened for HCC than older males [27]; another study on overall cancer screening differences among men and women showed that men were less likely to participate in cancer screening [28]. The latter is something that can be implied in this Georgian study sample as well; a higher proportion of women without HCV were diagnosed with HCC than males. However, the observed lower likelihood of males developing HCV-attributable HCC compared to females in this study may be due to the fact that males have a higher incidence of other risk factors for HCC [27].

In our study, similarly to the US study on HCV-attributable HCC, all attributable proportion calculations for the total population are low in predicting how much liver cancer can be attributed to HCV in different groups compared to the exposed population. However, this is plausible as there are several other predictors for HCC that could be present in the population (e.g., HBV infection, alcohol misuse, fatty liver disease, etc.) [23, 29].

### Limitations

In this study, the crude OR generated by the bivariate HCC sub-analysis is less than the crude OR for overall liver cancer. This underestimation could be attributable to the known classification limitations, and the likelihood that HCC cases may have been misclassified and thus included as controls. Depending on the number of cases underestimated/overestimated, the magnitude of effect observed in the HCC sub-analysis might be either under or overestimated in the study. Furthermore, only cases screened for HCV were included, which meant excluding around 34% of all liver cancer cases. It is likely that many cases of liver cancer were diagnosed at a later stage and the patients were never screened for HCV as they were put into hospice care [17], thus, potential, unidentified HCV-positive cases or HCV-negative cases were likely excluded from the study. This could either have underestimated or overestimated the magnitude of the association observed in the analysis. It should also be noted that the study’s youngest age group has very low rates of liver cancer and HCC, as HCC takes time to develop and is unlikely to be prevalent in the younger population. This explains the high variety of ORs in the age-stratified analysis and the comparatively high HCV-attributable proportion of HCC in the HCV-exposed population for the youngest age group. Thus, the results for this specific stratum should be interpreted with caution.

It is possible that HCV infection among those without confirmation testing is dependent on the outcome of liver cancer, thus higher among those with HCC as compared to those without HCC. If the prevalence of HCV infection is underestimated in the HCC population compared to the population without HCC, it can affect the magnitudes of effects observed in the study; we did however attempt to control for this through sensitivity analysis.

Cases were included in the study only if they had been screened for HCV, this meant excluding around 34% of all liver cancer cases in the years between 2015–2019. Considering the possible effect this might have had on the analysis is important. It is possible that many potential, unidentified HCV-positive cases, or, on the other hand, many HCV-negative cases were excluded from the study. This could either have underestimated or overestimated the magnitude of the association observed in the analysis.

In this study, controls were randomly selected to be representative of the exposure distribution in the general population; the prevalence of chronic HCV infection in the Georgian population (5.4%) [4] was the same as the prevalence of chronic HCV infection in the control sample. However, even though over 56% of the Georgian adult population was screened by 2020, and the general population has been widely offered screening, the possibility of selection bias regarding control selection should not be entirely disregarded; there is a possibility that the individuals who did end up getting screened by the year 2019 differ from those who did not by factors such as geographic location, socio-economic status, health conditions, etc.

Despite the efforts to ensure an adequate representation of the at-risk population, we did not perform demographic matching between cases and controls. While, as mentioned in the previous paragraph, controls were randomized and had the same prevalence of HCV as the actual general Georgian population, the lack of performed matching could be a source of bias and affect the interpretation of this study to an extent.

Another point of consideration is the classification of participants who tested positive for anti-HCV but negative for HCV RNA as HCV negative for the purpose of the analysis. These individuals may present cases of past chronic HCV infection who have previously had antiviral treatment. According to the registry data used for this study, neither such individuals had documented treatment, however, the possibility of them being positive for chronic HCV infection in the past still needs to be kept in mind.

Finally, since data for this study were extracted from registries, data on other potential confounders was limited. Thus, the analysis is missing some potential key confounders; unfortunately, these variables were hard to obtain for the study population and what information was available, was insufficient for analysis.

#### Significance of results

This study has highlighted the fact that a large proportion of liver cancer in the general population cannot be explained by HCV infection, and could be attributable to other factors such as HBV, alcohol consumption, fatty liver disease, etc. While the prevalence of HBV infection (2.9%) is lower in the Georgian population than the prevalence of HCV infection [4, 32], it is known that HBV causes more hepatitis-related liver cancer globally than HCV [3, 33]. Exploring the impact of HCV treatment on developing HCC could also be considered. The HCV elimination program offers the opportunity for recently-acquired HCV infections to be treated quickly, which could alter the effects of HCV on HCC and significantly reduce its burden in Georgia.

Another finding of this study was that patients with HCV in Georgia have a much higher risk of developing HCC than the general population. The surveillance of HCV-infected individuals (including those who have been cured but remain cirrhotic) for complications such as liver cancer should be prioritized in the country. The consensus worldwide regarding HCC surveillance is to screen cirrhotic patients using ultrasound with or without alpha-fetoprotein testing every six months [30]. Currently, the Georgian Ministry of Internally Displaced Persons from the Occupied Territories, Health, Labor, and Social Affairs only recommends HCC screening in cirrhotic patients, without details on time-frames of screening or any recommendations regarding HCV in general [31]. Given that every patient with viremic HCV infection in Georgia is assessed for cirrhosis before beginning treatment ^5–8^, more concrete guidelines for cirrhotic patients can be implemented to identify liver cancer as early as possible, affording a better chance of survival and a favorable outcome.

## Conclusion

This study has shown that HCV infection significantly increases the risk of liver cancer in the Georgian adult population and that females and those who are over the age of ≥ 50 years are more likely to develop it as a result of HCV infection. The high proportion of liver cancer and HCC attributable to HCV in the HCV-exposed population highlights the importance of the HCV elimination program in the country. Improved screening practices for cirrhotic patients could also be considered to detect early liver cancer cases. This study shows that there are potentially other, more significant predictors of liver cancer and HCC in the Georgian population that should be studied further to work towards decreasing the incidence of liver cancer in the country.

## Disclaimer

The findings and conclusions in this presentation are those of the author(s) and do not necessarily represent the views of the US Centers for Disease Control and Prevention/Agency for Toxic Substances and Disease Registry.

## Data Availability

The data that support the findings of this study are available from the Ministry of Internally Displaced Persons from the Occupied Territories, Labor, Health and Social Affairs of Georgia but restrictions apply to the availability of these data, which were used under license for the current study, and so are not publicly available. Data are however available from the authors upon reasonable request and with permission of the Ministry of Internally Displaced Persons from the Occupied Territories, Labor, Health and Social Affairs of Georgia.

## Footnotes

1 According to the HCV programmatic data.

## References

[1] World Health Organization. Hepatitis C. World Health Organization. Available from: https://www.who.int/news-room/fact-sheets/detail/hepatitis-c. Cited 2023 Apr 26.

[2] El-Serag HB. Epidemiology of viral hepatitis and hepatocellular carcinoma. Gastroenterology. 2012;142(6):1264–73. 10.1053/j.gastro.2011.12.061. 22537432 10.1053/j.gastro.2011.12.061 PMC3338949

[3] Maucort-Boulch D, de Martel C, Franceschi S, Plummer M. Fraction and incidence of liver cancer attributable to hepatitis B and C viruses worldwide. Int J Cancer. 2018;142(12):2471–7. 10.1002/ijc.31280. 29388206 10.1002/ijc.31280

[4] Hagan LM, Kasradze A, Salyer SJ, Gamkrelidze A, Alkhazashvili M, Chanturia G, et al. Hepatitis C prevalence and risk factors in Georgia, 2015: setting a baseline for elimination. BMC Public Health. 2019;19(Suppl 3):609. 10.1186/s12889-019-6784-3. 32326913 10.1186/s12889-019-6784-3 PMC6696670

[5] Averhoff F, Shadaker S, Gamkrelidze A, Kuchuloria T, Gvinjilia L, Getia V, et al. Progress and challenges of a pioneering hepatitis C elimination program in the country of Georgia. J Hepatol. 2020;72(4):680–7. 10.1016/j.jhep.2019.11.019. 31811882 10.1016/j.jhep.2019.11.019 PMC7418146

[6] Nasrullah M, Sergeenko D, Gamkrelidze A, Averhoff F. HCV elimination — lessons learned from a small Eurasian country. Georgia Nat Rev Gastroenterol Hepatol. 2017;14(8):447–8. 10.1038/nrgastro.2017.100. 28743936 10.1038/nrgastro.2017.100

[7] Tsertsvadze T, Gamkrelidze A, Chkhartishvili N, et al. Three Years of Progress Toward Achieving Hepatitis C Elimination in the Country of Georgia, April 2015–March 2018. Clin Infect Dis. 2019;71(5):1263–8. 10.1093/cid/ciz956. 10.1093/cid/ciz956 PMC7484896 31563938

[8] Mitruka K, Tsertsvadze T, Butsashvili M, et al. Launch of a Nationwide Hepatitis C Elimination Program-Georgia, April 2015. MMWR Morb Mortal Wkly Rep. 2015;64(28):753–7. 10.15585/mmwr.mm6428a2. 26203628 10.15585/mmwr.mm6428a2 PMC4584859

[9] Georgia Ministry of Internally Displaced Persons from the Occupied Territories L Health, and Social Affairs. Strategic Plan for the Elimination of Hepatitis c Virus in Georgia, 2016–2020.2017. https://www.moh.gov.ge/uploads/files/2017/akordeoni/failebi/Georgia_HCV_Elimination_Strategy_2016-2020.pdf.

[10] National Center for Disease Control and Public Health. Hepatitis C virus elimination progress report Georgia, 2015–2017. 2018. Available from: https://test.ncdc.ge/Pages/User/News.aspx?ID=7992bc6f-cb29-465e-9592-f8bf8402f106

[11] Harris AM, Chokoshvili O, Biddle J, et al. An evaluation of the hepatitis C testing, care and treatment program in the country of Georgia’s corrections system, December 2013 – April 2015. BMC Public Health. 2019;19(3):466. 10.1186/s12889-019-6783-4. 32326938 10.1186/s12889-019-6783-4 PMC6696696

[12] Shadaker S, Nasrullah M, Gamkrelidze A, et al. Screening and linkage to care for hepatitis C among inpatients in Georgia’s national hospital screening program. Prev Med. 2020;138: 106153. 10.1016/j.ypmed.2020.106153. 32473265 10.1016/j.ypmed.2020.106153 PMC7440391

[13] Gamkrelidze A, Gabunia T, Turdziladze A, Khonelidze I, Tsereteli M, Getia V, et al. Progress in hepatitis C testing as part of the hepatitis C elimination programme in Georgia. EMJ Hepatol [Internet]. 2020;8(Suppl 2):36-7. [cited 2024 Sep 17]. Available from: https://www.emjreviews.com/hepatology/abstract/progress-in-hepatitis-c-testing-as-part-of-the-hepatitis-c-elimination-programme-in-georgia/.

[14] Averhoff F, Lazarus JV, Sergeenko D, et al. Excellence in viral hepatitis elimination - Lessons from Georgia. J Hepatol. 2019;71(4):645–7. 10.1016/j.jhep.2019.06.026. 31356831 10.1016/j.jhep.2019.06.026

[15] Abesadze N. History of cancer registration in Georgia. Cauc J Health Sci Public Health. 2018;2(2):88–92.

[16] World Health Organization. ICD-10: International Statistical Classification of Diseases and Related Health Problems : Tenth Revision, 2nd Ed. World Health Organisation. 2nd ed.; 2004.

[17] Aslanikashvili A. HCV-attributable hepatocellular carcinoma among persons with hepatobiliary cancer diagnoses in Georgia: 2015–2016. Presentation presented at 4th Annual Hepatitic C Technical Advisory Group Meeting. Tbilisi, Georgia: 2018.

[18] Gamkrelidze A, Gabunia T, Turdziladze A, Khonelidze I, Tsereteli M, Getia V, et al. Progress in hepatitis C testing as part of the hepatitis C elimination program in Georgia. In: EASL. Abstracts. [Internet]. Vienna: International Liver Congress; 2019. [cited 2024 Sep 17]. Available from: https://easl.eu/wp-content/uploads/2020/12/EASL-ILC2019-AbstractBook.pdf.

[19] Bozdogan H. Model Selection and Akaike’s Information Criterion (AIC): The General Theory and Its Analytical Extensions. Psychometrika. 1987;52(3):345–70. 10.1007/BF02294361.

[20] Cole P, MacMahon B. Attributable risk percent in case-control studies. J Epidemiol Community Health. 1971;25(4):242–4. 10.1136/jech.25.4.242. 10.1136/jech.25.4.242 PMC478665 5160433

[21] Jaquet A, Tchounga B, Tanon A, et al. Etiology of hepatocellular carcinoma in West Africa, a case-control study. Int J Cancer. 2018;143(4):869–77. 10.1002/ijc.31393. 29569722 10.1002/ijc.31393 PMC6041181

[22] Mahale P, Torres H, Kramer J, et al. Hepatitis C virus infection and the risk of cancer among elderly US adults: A registry-based case-control study. Cancer. 2017;123(7):1202–11. 10.1002/cncr.30559. 28117886 10.1002/cncr.30559 PMC6295146

[23] Makarova-Rusher OV, Altekruse SF, McNeel TS, et al. Population attributable fractions of risk factors for hepatocellular carcinoma in the United States. Cancer. 2016;122(11):1757–65. 10.1002/cncr.29971. 26998818 10.1002/cncr.29971 PMC5548177

[24] Kohi MP. Gender-Related Differences in Hepatocellular Carcinoma: Does Sex Matter? J Vasc Interv Radiol. 2016;27(9):1338–41. 10.1016/j.jvir.2016.06.035. 27566425 10.1016/j.jvir.2016.06.035

[25] Naugler WE, Sakurai T, Kim S, et al. Gender disparity in liver cancer due to sex differences in MyD88-dependent IL-6 production. Science. 2007;317(5834):121–4. 10.1126/science.1140485. 17615358 10.1126/science.1140485

[26] Asahina Y, Tsuchiya K, Tamaki N, et al. Effect of aging on risk for hepatocellular carcinoma in chronic hepatitis C virus infection. Hepatology. 2010;52(2):518–27. 10.1002/hep.23691. 20683951 10.1002/hep.23691

[27] Wu EM, Wong LL, Hernandez BY, et al. Gender differences in hepatocellular cancer: disparities in nonalcoholic fatty liver disease/steatohepatitis and liver transplantation. Hepatoma Res. 2018;4:66. 10.20517/2394-5079.2018.87. 30687780 10.20517/2394-5079.2018.87 PMC6347119

[28] Davis J, Buchanan K, Katz R, Green B. Gender Differences in Cancer Screening Beliefs, Behaviors, and Willingness to Participate. Am J Mens Health. 2011;6(3):211–7. 10.1177/1557988311425853. 22071507 10.1177/1557988311425853 PMC3776317

[29] Ringehan M, McKeating J, Protzer U. Viral hepatitis and liver cancer. Philosophical Transactions of the Royal Society B: Biological Sciences. 2017;372(1732):20160274. 10.1098/rstb.2016.0274. 10.1098/rstb.2016.0274 PMC5597741 28893941

[30] Kasradze A, Shadaker S, Kuchuloria T, et al. The burden and epidemiology of hepatitis B and hepatitis D in Georgia: findings from the national seroprevalence survey. Public Health. 2020;185:341–7. 10.1016/j.puhe.2020.06.024. 32738575 10.1016/j.puhe.2020.06.024 PMC7467099

[31] Hiotis SP, Rahbari NN, Villanueva GA, et al. Hepatitis B vs. hepatitis C infection on viral hepatitis-associated hepatocellular carcinoma. BMC Gastroenterol. 2012;12((1):64. 10.1186/1471-230X-12-64. 22681852 10.1186/1471-230X-12-64 PMC3407024

[32] Yilmaz N, Yilmaz UE, Suer K, Goral V, Cakir N. Screening for hepatocellular carcinoma: summary of current guidelines up to 2018. Hepatoma Res. 2018;4:46. 10.20517/2394-5079.2018.49.

[33] Georgia Ministry of internally displaced persons from the occupied territories, health, and social affairs. ღვიძლის არაალკოჰოლური ცხიმოვანი დაავადების დიაგნოსტიკა და მართვა: კლინიკური მდგომარეობის მართვის სახელმწიფო სტანდარტი (პროტოკოლი). 2018. https://moh.gov.ge/ka/guidelines/.

